# Prevalence and factors associated with food insecurity in Latin America and the Caribbean during the first wave of the COVID-19 pandemic

**DOI:** 10.1016/j.heliyon.2021.e08091

**Published:** 2021-09-30

**Authors:** Vicente A. Benites-Zapata, Diego Urrunaga-Pastor, Mayra L. Solorzano-Vargas, Percy Herrera-Añazco, Angela Uyen-Cateriano, Guido Bendezu-Quispe, Carlos J. Toro-Huamanchumo, Adrian V. Hernandez

**Affiliations:** a Universidad San Ignacio de Loyola, Unidad para la Generación y Síntesis de Evidencias en Salud, Lima, Peru; b Red Internacional en Salud Colectiva y Salud Intercultural, México, Mexico; c Universidad San Ignacio de Loyola, Doctorado de Nutrición y Alimentos, Lima, Peru; d Universidad Científica del Sur, Lima, Peru; e Instituto de Evaluación de Tecnologías en Salud e Investigación – IETSI, EsSalud, Lima, Peru; f Universidad Privada San Juan Bautista, Lima, Peru; g Medecins Sans Frontieres, Health Politics, Brussels, Belgium; h Universidad Privada Norbert Wiener, Centro de Investigación Epidemiológica en Salud Global, Lima, Peru; i Clínica Avendaño, Unidad de Investigación Multidisciplinaria, Lima, Peru; j Health Outcomes, Policy, and Evidence Synthesis (HOPES) group, University of Connecticut School of Pharmacy, Storrs, CT, USA; k Universidad San Ignacio de Loyola, Unidad de Revisiones Sistemáticas y Metaanálisis, Guías de Práctica Clínica y Evaluaciones Tecnológicas Sanitarias, Lima, Peru

**Keywords:** Food insecurity, COVID-19, SARS-CoV-2, Latin America

## Abstract

**Objective:**

We assessed the prevalence of food insecurity (FI) and its associated factors in Latin American and the Caribbean (LAC) early during the COVID-19 pandemic.

**Methods:**

We performed secondary data analysis of a survey conducted by Facebook and the University of Maryland. We included adults surveyed from April to May 2020. FI was measured by concerns about having enough to eat during the following week. Sociodemographic, mental health, and COVID-19-related variables were collected. We performed generalized Poisson regressions models considering the complex sampling design. We estimated crude and adjusted prevalence ratios with their 95% confidence intervals.

**Results:**

We included 1,324,272 adults; 50.5% were female, 42.9% were under 35 years old, 78.9% lived in a city, and 18.6% had COVID-19 symptoms. The prevalence of food insecurity in LAC was 75.7% (n = 1,016,841), with Venezuela, Nicaragua, and Haiti with 90.8%, 86.7%, and 85.5%, respectively, showing the highest prevalence. Gender, area of residence, presence of COVID-19 symptoms, and fear of getting seriously ill or that a family member gets seriously ill from COVID-19 were associated with a higher prevalence of food insecurity. In contrast, increasing age was associated with a lower prevalence.

**Conclusion:**

The prevalence of food insecurity during the first stage of the COVID-19 pandemic in LAC was high and was associated with sociodemographic and COVID-19-related variables.

## 1 Introduction

The COVID-19 pandemic has spread worldwide, causing a social, economic, and health impact [1]. To date, the pandemic has claimed more than 2 million lives worldwide. More than 500 thousand deaths have been reported in Latin America and the Caribbean (LAC), with Brazil, Mexico and Colombia having the highest number of deaths per day [2, 3]. Prior to the pandemic, these countries already faced challenges, such as low economic growth, informality, poverty, inequality, vulnerability to natural disasters and the effects of climate change, which were exacerbated by the pandemic and further limited their health response capacity [1].

While the full impact of COVID-19 on the global economy is still uncertain, the health crisis has led to an economic slowdown that may increase food insecurity (FI). FI is a complex and dynamic process, it could be defined as uncertain or limited access to enough nutritious food for an active and healthy life [4]. The pandemic generated concern about food availability, especially in vulnerable populations with fewer resources to face unemployment, rising prices, and the low availability of food [5, 6]. Previously, the FI situation in low- and middle-income countries was alarming, with an estimated average of 821 million people undernourished globally between 2016 and 2018 [5,6]. Since the beginning of the pandemic, control measures to limit the spread of the virus included movement restrictions at various international, regional and national levels, causing severe disruption in supply chains and exacerbating the impact of the crisis, and having an important effect on the provision of medical supplies and food [7]. This disruption affected the distribution, availability and access to food and generated an increase in prices and less access to healthy food and state food programs [8, 9].

LAC is the most unequal region in the world, and before the pandemic, it already faced low levels of economic growth and high levels of labour informality. In addition, a fall of 9.1% in the regional gross domestic product (GDP) was estimated in 2020 due to the effects of the pandemic [1]. FI is added to this problem since it is estimated that of the 2 billion people who suffer from FI in the world, 205 million are in LAC [10]. Although Africa concentrates the highest levels of FI worldwide, the increase in LAC has been faster: rising from 22.9% in 2014 to 31.7% in 2019, mainly due to the increase in FI in South America [11].

Identifying modifiable factors associated with FI can serve as a basis for designing strategies that mitigate the consequences of disruptions caused by health crises, such as the pandemic and the consequent FI, and that strengthen regional food systems, making them more sustainable and sustainable. Although several studies have evaluated FI during the pandemic, the majority correspond to high- or low-income countries with socioeconomic and cultural characteristics that do not allow comparisons with our continent [12, 13, 14, 15, 16, 17, 18, 19, 20]. In LAC, studies in Brazil, Mexico, and Peru have been published [21, 22, 23]: however, these have been carried out in small samples and did not evaluate the influence of different sociodemographic factors. Therefore, the objective of this research was to know the prevalence and factors associated with FI in LAC countries during the early stage of quarantine due to COVID-19.

## 2 Methods

### 2.1 Design and study area

We carried out a secondary data analysis of a database generated by Facebook (Facebook, Inc) and the University of Maryland. These institutions performed a survey which objective was to collect relevant information related to the COVID-19 pandemic worldwide. The survey was divided into the following modules: sociodemographic characteristics, mental health, COVID-19 symptoms and contact reporting, economic and FI. This survey has been applied since April 2020 and was translated and applied to the participants in the main language of each country [24]. The selection of the surveyed participants was random, within the sample frame of Facebook users according to geographic region and country (each participant included had a weight according to the region and country in which they answered the survey). Daily, a certain number of people (variable according to each day) were invited to answer the survey. In case people declined or skipped the invitation, Facebook invited someone else within the same geographic area and that has not responded to the survey within the last eight weeks. This survey has been used to develop previous studies [25], where more information on the methodology is described.

### 2.2 Population and area

The survey was applied to adults (aged 18 and over) who used the social network Facebook. LAC adults who responded to the survey were included and totalled 1,440,586 participants. Participants without data related to the variables of interest were excluded, and therefore, 1,324,272 adults from 20 LAC countries were analyzed. The responses to the survey between April 23 and May 23, 2020, were included.

### 2.3 Variables and procedures

#### 2.3.1 Dependent variable: food insecurity

We evaluated FI using: "How worried are you about having enough to eat in the next week?" which is a question that allows four different answers: very worried, somewhat worried, not too worried, not worried at all. Later we dichotomized this variable as food security when the answer was “not worried at all”, while the other three were considered FI.

#### 2.3.2 Independent variables

##### 2.3.2.1 Sociodemographic variables

We evaluated the following variables: gender (categorized as male, female and non-binary), age (presented as 18 to 24, 25 to 34, 35 to 44, 45 to 54, 55 to 64, 65 to 74, and 75 or more), area of residence (rural area, village, town, and city) and if the participant currently worked outside their home (no, yes).

#### 2.3.2.2 Suspicious COVID-19 symptomatology and adherence to community mitigation strategies

We defined suspicious COVID-19 symptoms as the presence of more than two symptoms compatible with an acute condition of COVID-19 [26]. These included: fever, cough, muscle pain, loss of smell, shortness of breath, chest pain, sore throat, eye pain, coryza, tiredness, nausea and headache.

We evaluated adherence to the following community mitigation strategies: physical distancing, hands washing, and mask use. We considered adherence to physical distancing when participants denied any type of direct contact (such as shaking hands, touching, kissing, hugging) for a period longer than one minute in the last 24 h and maintained a distance greater than two meters from any person who does not live in the same household. Likewise, we considered handwashing compliance when participants reported at least one hand wash after leaving home in the last seven days. We defined mask compliance when a mask was used in public (at least at some point) during the last seven days.

#### 2.3.2.3 Mental health

We evaluated depressive symptoms based on one adapted question from the Kessler Psychological Distress Scale (K10): "In the past 7 days, about how often did you feel depressed?", with five categories (all of the time, most of the time, some of the time, a little of the time, none of the time) [27]. Then, we dichotomized the variable as absence of depressive symptoms when the answer was "none of the time" and presence of depressive symptoms as any of the four remaining options.

We evaluated anxiety symptoms using one adapted question from the Kessler Psychological Distress Scale (K10): "During the last 7 days, how often did you feel so nervous that nothing could calm you down?" This question allowed five possible answers (all of the time, most of the time, some of the time, a little of the time, none of the time) [27]. Then, we dichotomized the variable as the absence of anxious symptoms if they answered, “none of the time” and the presence of anxious symptoms if they answered any of the other four options.

We assessed the fear of getting seriously ill or that a family member gets seriously ill from COVID-19 with the subsequent question: "How worried are you that you or someone in your immediate family might become seriously ill from coronavirus (COVID-19)?". This allowed four responses (very worried, somewhat worried, not too worried, not worried at all). Then, we considered “not worried at all” as the absence of fear of getting seriously ill or that any member of the participant's family will get ill with COVID-19, and the other three options defined the presence of this condition.

### 2.4 Statistical analysis

We imported our database into the statistical package STATA v14.0 (StataCorp, TX, USA) from a Microsoft Excel 2010 format. For the statistical analyses, we used the svy command to take in count the complex sampling employed by the survey.

Qualitative variables were described with absolute frequencies and proportions considering the survey complex sampling and their 95% confidence intervals (95%CI). For the bivariate analysis among the variables of interest and the FI, we used the Rao-Scott Chi-square test. The association between independent variables and FI was estimated using a generalized linear model from the Poisson family with a logarithmic link function. Then, we estimated crude and adjusted prevalence ratios (PR) with their respective 95%CI. Using a statistical approach in the adjusted model, we included the variables with a p-value <0.05 in the crude model. Finally, we evaluated collinearity among the variables in the adjusted model conceptually and using the inflation factor of the variance.

### 2.5 Ethical aspects

All the participants provided informed consent to participate before the start of the survey. The survey and its privacy practices were reviewed by the Institutional Review Board of the University of Maryland. This institution allowed access through a database use agreement. No personal identifiers were taking into count during the download and analysis of the data.

## 3 Results

### 3.1 Characteristics of the study sample

We analyzed 1,324,272 LAC adults, of whom 50.5% (n = 723,545) were female, 42.9% (n = 722,070) were under 35 years of age, 78.9% (n = 1,041,369) lived in a city and 18.6% (n = 277,301) had symptoms of COVID-19. Fear of getting seriously ill or that a family member gets seriously ill from COVID-19 was reported by 92.3% (n = 1,238,859), while 46.6% (n = 670,861) had depressive symptoms and 28.6% (n = 379,214) reported working outside from home. FI was described by 75.7% (n = 1,016,841). The adults’ characteristics are displayed in Table 1.

**Table 1:** Characteristics of the study sample (n = 1,324,272; N = 11,392,965).

Characteristics	Total		
	Absolute frequency of participants surveyed	Weighted proportion according to each category	
	n	%	95%CI
Gender			
Male	586,275	48.1	47.7–48.5
Female	723,545	50.5	50.1–50.9
Non-binary	14,452	1.4	1.2–1.7
Age (years)			
18-24	313,345	18.1	17.3–18.8
25-34	408,725	24.8	24.1–25.5
35-44	280,296	18.7	18.4–19.0
45-54	177,459	18.7	18.4–19.1
55-64	103,325	11.1	10.7–11.5
65-74	35,127	7.4	6.9–8.0
75 years or older	5,995	1.2	1.1–1.3
Living area			
City	1,041,369	78.9	75.8–81.8
Town	183,959	13.8	11.5–16.5
Village or rural area	98,944	7.3	6.6–8.0
COVID-19 symptomatology			
No	1,046,971	81.4	80.5–82.3
Yes	277,301	18.6	17.7–19.5
Compliance with the CMS			
No	736,781	54.8	53.7–55.9
Yes	587,491	45.2	44.1–46.3
Fear of getting seriously ill or that a family member gets seriously ill from COVID-19			
No	85,413	7.7	7.0–8.5
Yes	1,238,859	92.3	91.5–93.0
Anxiety symptomatology			
No	691,608	55.3	54.7–55.9
Yes	632,664	44.7	44.1–45.3
Depressive symptomatology			
No	653,411	53.4	52.6–54.1
Yes	670,861	46.6	45.9–47.4
Currently works outside the house			
No	945,058	71.4	70.2–72.5
Yes	379,214	28.6	27.5–29.8
Food insecurity			
No	307,431	24.3	23.1–25.4
Yes	1,016,841	75.7	74.6–76.9

CMS: Community mitigation strategies; CI: Confidence intervals.

### 3.2 Bivariate analysis according to food insecurity

We found statistically significant differences between FI and the covariates, except for the adherence to community mitigation strategies (p = 0.347). These results are described in the Table 2.

**Table 2:** Descriptive and bivariate analysis of the study sample characteristics according to food insecurity (n = 1,324,272; N = 11,392,965).

Characteristics	Food insecurity						p value
	Yes			No			
	Absolute frequency of participants surveyed	Weighted proportion according to each category		Absolute frequency of participants surveyed	Weighted proportion according to each category		
	n	%	95%CI	n	%	95%CI	
Gender							<0.001
Male	442,632	74.8	73.6–75.9	143,643	25.2	24.1–26.4	
Female	562,755	76.6	75.4–77.7	160,790	23.4	22.3–24.6	
Non-binary	11,454	77.1	74.0–80.0	2,998	22.9	20.0–26.0	
Age (years)							<0.001
18-24	259,885	83.7	82.8–84.6	53,460	16.3	15.4–17.2	
25-34	329,009	82.5	81.6–83.3	79,716	17.5	16.7–18.4	
35-44	216,579	79.6	78.7–80.5	63,717	20.4	19.5–21.3	
45-54	127,797	74.3	73.4–75.3	49,662	25.7	24.7–26.6	
55-64	64,028	64.9	63.5–66.2	39,297	35.1	33.8–36.5	
65-74	17,082	49.6	47.9–51.3	18,045	50.4	48.7–52.1	
75 years or older	2,461	40.5	37.7–43.4	3,534	59.5	56.6–62.3	
Living area							<0.001
City	792,561	74.9	73.8–76.0	248,808	25.1	24.0–26.2	
Town	146,769	79.8	78.5–81.1	37,190	20.2	18.9–21.5	
Village or rural area	77,511	76.6	75.3–77.7	21,433	23.4	22.3–24.7	
COVID-19 symptomatology							<0.001
No	786,662	74	72.7–75.3	260,309	26	24.7–27.3	
Yes	230,179	83.1	82.4–83.9	47,122	16.9	16.1–17.6	
Compliance with the three CMS							0,347
No	567,400	75.8	74.7–76.9	169,381	24.2	23.1–25.3	
Yes	449,441	75.6	74.3–76.9	138,050	24.4	23.1–25.7	
Fear of getting seriously ill or that a family member gets seriously ill from COVID-19							<0.001
No	39,043	44.1	42.6–45.7	46,370	55.9	54.3–57.4	
Yes	977,798	78.4	77.0–79.7	261,061	21.6	20.3–23.0	
Anxiety symptomatology							<0.001
No	486,858	69.1	67.8–70.3	204,750	30.9	29.7–32.2	
Yes	529,983	84	82.8–85.1	102,681	16	14.9–17.2	
Depressive symptomatology							<0.001
No	454,855	68.4	67.2–69.6	198,556	31.6	30.4–32.8	
Yes	561,986	84.1	82.8–85.3	108,875	15.9	14.7–17.2	
Currently works outside the house							<0.001
No	722,343	74.9	73.6–76.2	222,715	25.1	23.8–26.4	
Yes	294,498	77.7	76.8–78.7	84,716	22.3	21.3–23.2	

CMS: Community mitigation strategies; CI: Confidence intervals.

### 3.3 Prevalence of food insecurity by country

**Figure 1:**
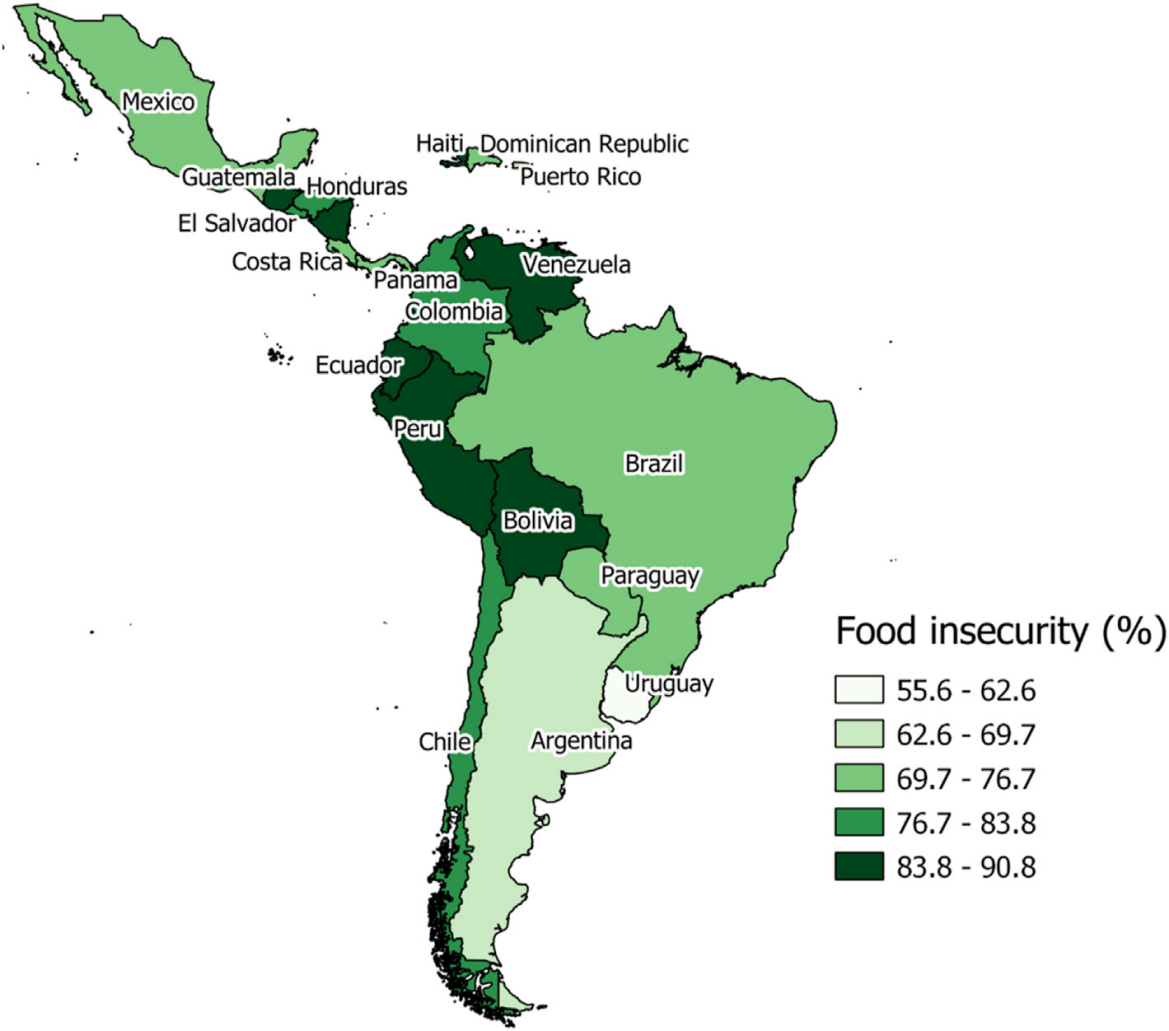
Prevalence (%) of food insecurity in Latin American and the Caribbean.

The highest prevalence of FI belonged to Venezuela (90.8%), Nicaragua (86.7%), Haiti (85.5%), Ecuador (85.1%), Bolivia (84.2%), and Peru (83.9%). On the other hand, those with a lower prevalence were Uruguay (55.6%), Puerto Rico (67.2%), and Argentina (68.6%) (Figure 1 and Table 3).

**Table 3:** Proportion of food insecurity according to countries included in the study sample.

Countries	Food insecurity			
	No		Yes	
	%	95%CI	%	95%CI
Argentina	31.4	29.8–33.1	68.6	66.9–70.2
Bolivia	15.8	14.5–17.2	84.2	82.8–85.6
Brazil	27.5	26.7–28.6	72.5	71.4–73.4
Chile	23.0	22.2–23.7	77.0	76.3–77.8
Colombia	23.2	21.6–24.8	76.8	75.2–78.4
Costa Rica	29.6	26.6–32.9	70.4	67.1–73.4
Dominican Republic	24.9	23.0–26.9	75.1	73.1–77.0
Ecuador	14.9	13.9–16.0	85.1	84.0–86.1
El Salvador	16.7	15.8–17.7	83.3	82.3–84.2
Guatemala	16.2	15.4–17.2	83.8	82.8–84.6
Haiti	14.5	10.2–20.2	85.5	79.8–89.8
Honduras	18.4	17.3–19.4	81.6	80.6–82.7
Mexico	24.1	22.3–25.9	75.9	74.1–77.7
Nicaragua	13.3	12.0–14.8	86.7	85.2–88.0
Panama	26.7	23.7–30.0	73.3	70.0–76.3
Paraguay	26.8	25.6–28.1	73.2	71.9–74.4
Peru	16.1	14.5–17.9	83.9	82.1–85.5
Puerto Rico, U.S.	32.8	29.2–36.7	67.2	63.3–70.8
Uruguay	44.4	41.0–47.8	55.6	52.2–59.0
Venezuela	9.2	8.2–10.3	90.8	89.7–91.8

CI: Confidence intervals.

### 3.4 Factors associated with food insecurity

In the adjusted regression model, the genders with a higher prevalence of FI were female (aPR = 1.01; 95%CI: 1.01–1.02; p < 0.001) and non-binary (aPR = 1.05; 95%CI: 1.02–1.08; p = 0.006) compared with males. Participants who lived in a rural area or village (aPR = 1.03; 95%CI: 1.02–1.04; p < 0.001) or in a town (aPR = 1.06; 95%CI: 1.04–1.07; p < 0.001) compared to those who lived in a city presented a higher prevalence of FI. Likewise, having COVID-19 symptoms (aPR = 1.06; 95%CI: 1.05–1.07; p < 0.001) and being afraid of getting seriously ill or that a family member gets seriously ill from COVID-19 (aPR = 1.71; 95%CI: 1.67–1.75; p < 0.001) were associated with a greater prevalence of FI. Finally, age from 25 to 34 years old (aPR = 0.99; 95%CI: 0.98–0.99; p < 0.001), 35–44 (aPR = 0.96; 95%CI: 0.96–0.97; p < 0.001), 45–54 (aPR = 0.91; 95%CI: 0.90–0.91; p < 0.001), 55–64 (aPR = 0.80; 95%CI: 0.79–0.81; p < 0.001), 65–74 (aPR = 0.62; 95%CI: 0.60–0.64; p < 0.001) and 75 years and over (aPR = 0.51; 95%CI: 0.48–0.55; p < 0.001) were associated with a minor prevalence of FI in comparison to ages between 18-24 years old (Table 4).

**Table 4:** Crude and adjusted generalized linear models of Poisson family with link log to evaluate the factors associated to food insecurity in the study sample.

Variables	Crude			Adjusted		
	cPR	95%CI	p value	aPR	95%CI	p value
Gender						
Male	Reference	-	-	Reference	-	-
Female	1.02	1.02–1.03	<0.001	1.01	1.01–1.02	<0.001
Non-binary	1.03	1.00–1.07	0.062	1.05	1.02–1.08	0.006
Age (years)						
18-24	Reference	-	-	Reference	-	-
25-34	0.99	0.98–0.99	<0.001	0.99	0.98–0.99	<0.001
35-44	0.95	0.95–0.96	<0.001	0.96	0.96–0.97	<0.001
45-54	0.89	0.88–0.90	<0.001	0.91	0.90–0.91	<0.001
55-64	0.78	0.76–0.79	<0.001	0.80	0.79–0.81	<0.001
65-74	0.59	0.58–0.61	<0.001	0.62	0.60–0.64	<0.001
75 years or older	0.48	0.45–0.52	<0.001	0.51	0.48–0.55	<0.001
Living area						
City	Reference	-	-	Reference	-	-
Town	1.07	1.05–1.08	<0.001	1.06	1.04–1.07	<0.001
Village or rural area	1.02	1.01–1.03	<0.001	1.03	1.02–1.04	<0.001
COVID-19 symptomatology						
No	Reference	-	-	Reference	-	-
Yes	1.12	1.11–1.14	<0.001	1.06	1.05–1.07	<0.001
Compliance with the three CMS						
No	Reference	-	-			
Yes	0.99	0.99–1.00	0.353	Not included		
Fear of getting seriously ill or that a family member gets seriously ill from COVID-19						
No	Reference	-	-	Reference	-	-
Yes	1.78	1.73–1.82	<0.001	1.71	1.67–1.75	<0.001
Anxiety symptomatology						
No	Reference	-	-			
Yes	1.22	1.21–1.22	<0.001	Not included∗		
Depressive symptomatology						
No	Reference	-	-			
Yes	1.23	1.22–1.24	<0.001	Not included∗		
Currently works outside the house						
No	Reference	-	-	Reference	-	-
Yes	1.04	1.03–1.05	<0.001	1.00	1.00–1.01	0.297

CMS: Community mitigation strategies; CI: Confidence intervals; cPR: Crude prevalence ratio; aPR: Adjusted prevalence ratio.

∗ Not included due to collinearity with fear of becoming seriously ill or that a family member becomes seriously ill from COVID-19.

## 4 Discussion

This study aimed to identify the factors associated with FI in LAC. We found that the prevalence of FI during the first stage (April–May 2020) of the COVID-19 pandemic in LAC was high. Factors such as gender, the area of residence, suspicious COVID-19 symptoms, and the fear of getting seriously ill or that a family member gets seriously ill from COVID-19 were associated with a higher probability of FI. On the contrary, an increase in age was associated with a lower prevalence.

Three out of four people in LAC suffered from FI during the first stage of the pandemic. A study carried out in the United States showed a 13% increase in FI during the first month after the onset of the pandemic, while in Italy, another study showed an increase of 16.2% six months after the beginning of the outbreak [15, 16]. Another study conducted in Australia found a prevalence of FI of 26% at the beginning of the pandemic, while a study in Iran showed a decrease in severe FI by three percentage points over a short period and was associated with purchasing and warehousing of food due to government policies of isolation and closure of some production plants [18]. Studies carried out in Bangladesh, Iran and Jordan showed results similar to those of our study [17, 19, 20]. At the LAC level, previous research corroborates our results, indicating that in the first stage of the pandemic, two favelas located in Brazil [21] and a sector of the population of Peru [23] had FI, while in Mexico [22], one study showed that FI was found at all socioeconomic levels. Our study, however, described a higher prevalence, which may be related to the measurement of FI at the beginning of quarantine in many LAC countries. The application of control measures as movement restrictions to limit the virus spread could have provoked severe disruption in food supply chains in a region with a high prevalence of FI before the pandemic [7]. Similarly, it may be related to aspects described in other countries during the pandemic, such as unemployment, business close, and income reduction in the population [28].

Female and non-binary genders were found to have higher FI compared to males. The 2020 Food Safety and Nutrition Report found a higher prevalence of FI in women than in men [11]. This difference was statistically significant globally in 2019, and especially in LAC, where the same difference was found for all the years of analysis and in which the gender gap in access to food also increased from 2018 to 2019 [1]. A study carried out in the United States agrees with our results, and the authors associate their results with certain traditional roles in which women make home purchases. Likewise, a decrease in income was associated with having suffered from FI before the pandemic [12]. This study also evaluated non-binary gender, showing no significant differences. Furthermore, a study conducted in Canada, found that mothers showed greater concern about limited financial resources due to job losses, reduced working hours and the overload of domestic work due to the closure of schools [29].

Our finding of a higher FI in rural areas may be related to the predominance of informal businesses and situations of extreme poverty in these areas, despite having easier access to self-produced food [23]. On the other hand, it has been reported that during the pandemic in Bangladesh, urban households had a higher prevalence of severe FI (42%) than rural households (15%). It was also noted that urban households were more likely to apply for financial mitigation techniques and access to food than their rural counterparts [19]. Other studies in this country showed that farmers in rural areas had serious difficulties in selling and transporting their products because of higher transport costs and a shortage of workers. Then, despite having higher food prices in the cities, the prices of the products in markets located in rural areas fell by 17%–70%, and farmers experienced large amounts of food waste, causing financial losses in the agricultural-based rural economy [30]. On the other hand, a report by the Food and Agriculture Organization (FAO) on the impact of COVID-19 on food markets in Bangladesh pointed out that fresh food stores of fruits, fresh vegetables, and meat were closed in urban areas, and average food prices increased by up to 20% [31]. FI evaluates the quantity and quality of food, and then, it is related to food preservation. In this way, in rural areas, FI could be more affected due to the absence of refrigerators. A previous study in Bangladesh found that 41.2% of the population did not have a refrigerator, and this group had a higher probability of FI [28]. Likewise, in 2017, it was described that 49% of the Peruvian population had a refrigerator, but only 7.7% resided in rural areas [32].

`When FI becomes manifest after natural disasters, an association has also been found with the population of rural environments because this population has fewer economic resources and receives little government aid, as well as less possibility of accessing food banks or assistance programs [33, 34, 35]. Most LAC countries have a large workforce in agriculture and livestock, and these sectors have been directly affected by the pandemic [36]. In addition, agricultural workers depend on the sale of their production, and their income is reduced compared to those who work in an urban area since they can adapt to remote work [37].

A gradual decrease in the prevalence of FI was found in relation to increasing age, being lower in participants older than 75 years. A previous study in Australia in the early stage of the pandemic also found that increasing age decreased the probability of FI and that FI decreased by 16% with each decade of difference. The young population was described as being the most affected since they received less income due to the reduction of temporary or part-time jobs [18]. Other studies carried out in the United States, Belgium, Jordan, and Italy reported similar results [13, 15, 16, 20]. It should be noted that given that an older adult in LAC have less use of digital platforms due to fear of complexity or difficulty in using their functions [38, 39, 40], and 42.9% of the people analyzed were under 35 years of age, the findings reported here are not fully representative of people of legal age from LAC.

Having COVID-19 symptoms was another factor associated with FI. The confinement measures taken as a precaution to avoid the spread of the virus in case of having symptoms associated with COVID-19 could explain this finding, with the restrictions in mobility to search for food increasing FI. On the other hand, concern about a relative getting infected was also associated with a higher prevalence of FI. A study conducted in Peru found that respondents with family members suspected of COVID-19 had a higher prevalence of FI [23]. This might be explained in that it is likely that the family member who became ill made the household purchases or generated part of the family income, implying the need to seek new sources of income to cover health and food expenses, as well as the redistribution of household tasks. In the United States, research showed that lower food security decreased the likelihood of requesting vacation days if a family member fell ill with COVID-19 [14].

The countries in which a higher frequency of FI was found have lower per capita incomes. In 2019, the country with the lowest GDP per capita in LAC was Haiti, a nation in which undernourishment is present in 48.2% of the population and has remained constant for the last ten years [41]. Likewise, the political-economic crisis in Venezuela has led to hyperinflation, which began at the end of 2017, with a subsequent increase in FI and undernourishment, the latter having a prevalence of 31.4% [41, 42, 43]. On the contrary, FI is lower in countries such as Uruguay, probably due to their high per capita income, low levels of inequality, indigence, and unemployment [44]. Due to work informality, during quarantine, people are likely to experience greater concern about getting food and therefore greater FI. In Latin America, almost 50% of the people are informal workers, most of whom are located in Central America [45]. In the context of COVID-19, many companies closed, and their workers were laid off. Furthermore, informal workers were affected by the confinement measures, ceasing to receive regular income. However, countries such as Uruguay implemented public policies to reduce informal employment, and its social security reforms focused on income transfers to the most vulnerable sectors with flexible access to net benefits in health and security against unemployment [46].

Some measures that governments have implemented to reduce FI include unemployment benefits, eliminating measures that restrict trade in essential products such as food, emergency food assistance programs, and the massive dissemination of nutritious and healthy diet options and accessible food [47]. The COVID-19 pandemic has affected the agricultural sector, and therefore, some public policies should be aimed at training farmers to use low-cost innovations for better production [48]. Other interventions are those based on social responsibility and solidarity of individuals or private companies. There are food banks in many countries. However, they often do not achieve the necessary impact because they are not driven by the government and lack regulations. The use of existing food assistance programs could be used as a platform to monitor FI [49]. These strategies should mainly be focused on the most vulnerable populations such as people living in extreme poverty, older adults, children, pregnant women, indigenous people, immigrants, people at high risk of contagion due to comorbidities, and families with numerous members that reside in rural areas.

Among the study's limitations, it should be pointed out the mode of FI measurement was based mainly on the concern for obtaining food, not evaluating aspects related to the quality of the food. The measurements used to evaluate depressive and anxious symptoms are part of the K10 scale. However, they have not been validated separately. Even so, they provide pertinent information for the study. Likewise, the cross-sectional design does not allow establishing causal relationships between the variables associated with FI. Another limitation is the use of a social network such as Facebook to carry out the survey, being that access to this service by population groups of extreme poverty would be limited. Thus, these groups are not represented in the results of this study. Nonetheless, the analysis of FI in LAC was carried out at the beginning of the pandemic in a large sample at the country level in a social network where four out of every five people in this region belong.

In conclusion, three out of four respondents in LAC reported FI at the start of the COVID-19 pandemic. The differences in the frequency of FI in the different countries are consistent with the level of poverty and work informality before the pandemic. We found sociodemographic and COVID-19 related factors associated with FI. Based on our findings, we can elaborate on some recommendations with public health implications. Governments should design policies to protect vulnerable populations such as rural areas habitants against FI, which may include employment programs, economic unemployment benefits, improvement in food distribution chains, and promoting state food programs and nutritional counselling.

## Declarations

### Author contribution statement

Vicente A. Benites-Zapata, Diego Urrunaga-Pastor: Conceived and designed the experiments; ​Performed the experiments; ​Analyzed ​and interpreted the data; Contributed reagents, materials, analysis tools or data; Wrote the paper.

Mayra L. Solorzano-Vargas, Percy Herrera-Añazco, Angela Uyen-Cateriano, Guido Bendezu-Quispe, Carlos J. Toro-Huamanchumo, Adrian V. Hernandez: Conceived and designed the experiments; Wrote the paper.

### Funding statement

This research did not receive any specific grant from funding agencies in the public, commercial, or not-for-profit sectors.

### Data availability statement

The authors do not have permission to share data.

### Declaration of interests statement

The authors declare no conflict of interest.

### Additional information

No additional information is available for this paper.
